# Expression Profiles of Circulating microRNAs in South African Type 2 Diabetic Individuals on Treatment

**DOI:** 10.3389/fgene.2021.702410

**Published:** 2021-09-08

**Authors:** Cecil J. Weale, Don M. Matshazi, Saarah F. G. Davids, Shanel Raghubeer, Rajiv T. Erasmus, Andre P. Kengne, Glenda M. Davison, Tandi E. Matsha

**Affiliations:** ^1^SAMRC/CPUT/Cardiometabolic Health Research Unit, Department of Biomedical Sciences, Faculty of Health and Wellness Science, Cape Peninsula University of Technology, Cape Town, South Africa; ^2^National Health Laboratory Service (NHLS), Division of Chemical Pathology, Faculty of Health Sciences, University of Stellenbosch, Cape Town, South Africa; ^3^Non-Communicable Diseases Research Unit, South African Medical Research Council, Cape Town, South Africa; ^4^Department of Medicine, University of Cape Town, Cape Town, South Africa

**Keywords:** South Africa, miR-30a-5p, miR-1299, miR-182-5p, miR-126-3p, treatment, type 2 diabetes

## Abstract

**Aim:** The influence of disease duration and anti-diabetic treatment on epigenetic processes has been described, with limited focus on interactions with microRNAs (miRNAs). miRNAs have been found to play key roles in the regulation of pathways associated with type 2 diabetes mellitus (T2DM), and expression patterns in response to treatment may further promote their use as therapeutic targets in T2DM and its associated complications. We therefore aimed to investigate the expressions of circulating miRNAs (miR-30a-5p, miR-1299, miR-182-5p, miR-30e-3p and miR-126-3p) in newly diagnosed and known diabetics on treatment, in South Africa.

**Methods:** A total of 1254 participants with an average age of 53.8years were included in the study and classified according to glycaemic status (974 normotolerant, 92 screen-detected diabetes and 188 known diabetes). Whole blood levels of miR-30a-5p, miR-1299, miR-182-5p, miR-30e-3p and miR-126-3p were quantitated using RT-qPCR. Expression analysis was performed and compared across groups.

**Results:** All miRNAs were significantly overexpressed in subjects with known diabetes when compared to normotolerant individuals, as well as known diabetics vs. screen-detected (*p*<0.001). Upon performing regression analysis, of all miRNAs, only miR-182-5p remained associated with the duration of the disease after adjustment for type of treatment (OR: 0.127, CI: 0.018–0.236, *p*=0.023).

**Conclusion:** Our findings revealed important associations and altered expression patterns of miR-30a-5p, miR-1299, miR-182-5p, miR-30e-3p and miR-126-3p in known diabetics on anti-diabetic treatment compared to newly diagnosed individuals. Additionally, miR-182-5p expression decreased with increasing duration of T2DM. Further studies are, however, recommended to shed light on the involvement of the miRNA in insulin signalling and glucose homeostasis, to endorse its use as a therapeutic target in DM and its associated complications.

## Introduction

MicroRNAs (miRNAs) are a family of short, noncoding RNA molecules, averaging 22 nucleotides in length, responsible for regulating gene expression by repressing the translation of messenger RNA (mRNA) molecules, as well as by destabilization of the mRNA molecules (Zhou et al., 2015; O’Brien et al., 2018). Since their discovery, miRNAs have been found to play key roles in the regulation of pathways associated with various diseases, including cancers (Liu et al., 2017; Zhang et al., 2019), cardiovascular diseases (Maciejak et al., 2018; Zhu et al., 2019), and diabetes mellitus (DM; Zampetaki et al., 2010; Massaro et al., 2019; Wang et al., 2019). These small noncoding transcripts have been shown to modulate insulin biosynthesis, pancreatic β-cell development and survival, as well as glucose and lipid metabolism (Mao et al., 2013). Investigations have illustrated altered expression levels of miRNAs, such as miR-30a-5p and miR-126-3p across different glycaemic states, suggesting their potential use as novel biomarkers for early detection of diabetes (Qin et al., 2017; Jiménez-Lucena et al., 2018a). With increased exploration of human miRNAs in the setting of disease, strategies for diagnosis have been the primary focus. However, the focus has progressively extended toward assessing miRNA expression levels and treatment of disease (Walayat et al., 2018).

The interactions of medications, such as metformin with epigenetic processes, have been detailed, including influences in histone modifications, as well as DNA methylation. DNA methylation-induced changes have been the most widely addressed, with reduced methylation due to metformin treatment reported at the insulin gene promoter in a beta-cell line cultured using high glucose concentrations (Ishikawa et al., 2015). Similarly, reduced methylation of transporter genes (SLC22A1, SLC22A3 and SLC47A1) was reported in the livers of diabetics on metformin therapy, compared to diabetics not receiving anti-diabetic medication (García-Calzón et al., 2017). Although not as widely investigated, key evidence surrounding the associations between DM therapies and miRNA expression also exists, with reports of metformin-induced alterations in miRNA expression in diabetic humans and mice, due to increased levels of DICER enzymes, which are essential in miRNA processing (Noren Hooten et al., 2016). Additionally, plasma miR-222 has been linked with insulin action, and positive associations have been identified with type 2 diabetes mellitus (T2DM; Coleman et al., 2013). In two separate studies, both insulin infusion and metformin treatment led to reduced circulating levels of the miRNA in patients with T2DM (Coleman et al., 2013; Ortega et al., 2014). In the same way, miRNA expressions aid in the mediation of these processes in disease development and progression, and changes in their expression in response to anti-diabetic treatment may pave the way for new therapeutic strategies. In view of this, we aimed to investigate the expression of a panel of miRNAs, previously demonstrating altered expressions (Matsha et al., 2018), in a South African population with T2DM, receiving either metformin treatment, insulin and/or both. Furthermore, we intended to assess the expression levels in association with the duration of T2DM since the date of first diagnosis. Our findings in addition to other investigations focused on miRNA dysregulation in T2DM development and progression may lay the foundation for new targets for disease management and therapy.

## Materials and Methods

### Ethical Approval

The study was approved by the Research Ethics Committees of the Cape Peninsula University of Technology and Stellenbosch University (NHREC: REC—230, 408–014 and N14/01/003, respectively). Ethical clearance for this sub-study was sought from and granted by the Cape Peninsula University of Technology Ethics Committee (CPUT/HW-REC 2019/H3). The study was conducted in accordance with the Declaration of Helsinki, and all participants voluntarily signed written informed consent after all the procedures were fully explained in their language of choice.

### Study Design

This study was cross-sectional in design, and data were obtained from the ongoing Cape Town Vascular and Metabolic Health (VMH) study, as previously described (Kengne et al., 2017). Data collection took place between April 2014 and November 2016, involving only South Africans from Cape Town. In a total of 1989 participants that were enrolled in the VMH study, 1,254 met the inclusion criteria for this study, including 92 screen-detected diabetes, 188 known diabetics on treatment and 974 normotolerant individuals. Similarly, a recently conducted cross-sectional investigation, sourced their sample from a much larger, previously reported cohort, with a total sample size of 218 test subjects (Prattichizzo et al., 2021a). With ours being larger, this then validates the size of the chosen sample in this study. Participants with unknown glucose tolerance status underwent a 75g oral glucose tolerance test in accordance with WHO guidelines (Alberti and Zimmet, 1998), and study procedures, such as anthropometric and blood pressure measurements, were also assessed.

Biochemical parameters were immediately analysed at an ISO 15189 accredited pathology practice (PathCare, Reference Laboratory, Cape Town, South Africa). Blood glucose levels (mmol/L) were determined using an enzymatic hexokinase method (Beckman AU, Beckman Coulter, South Africa), and HbA1c levels were determined by high-performance liquid chromatography (BioRad Variant Turbo, BioRad, South Africa). Serum insulin was assessed using a paramagnetic particle chemiluminescence assay (Beckman DXI, Beckman Coulter, South Africa). Serum high-density lipoprotein cholesterol (cholesterol HDL-S; mmol/L) was measured by enzymatic immune-inhibition – End Point (Beckman AU, Beckman Coulter, South Africa), and serum low-density lipoprotein cholesterol (cholesterol LDL-S; mmol/L) by enzymatic selective protection – End Point (Beckman AU, Beckman Coulter, South Africa) and serum triglycerides (triglycerides-S; mmol/L) were estimated by glycerol phosphate oxidase-peroxidase, End Point (Beckman AU, Beckman Coulter, South Africa). Ultrasensitive C-reactive protein (usCRP) was measured by Latex Particle immunoturbidimetry. Serum γ-glutamyltransferase (gamma GT-S) was measured using the International Federation of Clinical Chemistry and Laboratory Medicine standardized reagents on a Beckman AU (Beckman Coulter, South Africa). Serum cotinine was determined using the Competitive Chemiluminescent (Immulite 2000, Siemens, South Africa).

### Total RNA Isolation

Total RNA, including miRNAs, was isolated from 3ml of whole blood, which was collected in Tempus RNA tubes that were stored at −80°C before RNA isolation (Applied Biosystems). The MagMAX^™^ for Stabilized Blood Tubes RNA Isolation Kit was used to perform the RNA extraction, as per manufacturer’s specifications (Life Technologies, South Africa). RNA purity and integrity were then evaluated using a nanodrop (Nanodrop Technologies, Wilmington, United States), and only samples with a concentration>15ng/ml, and an OD (optical density) ratio ^A260^/_A280_>1.8, were deemed adequate for further processing.

### cDNA Conversion and Reverse Transcriptase qPCR

Subsequent RNA samples were then converted to cDNA, using the TaqMan Advanced miRNA cDNA Synthesis Kit, following manufacturer guidelines (Applied Biosystems, Thermo Fisher Scientific, South Africa). This protocol converts RNA to cDNA in four separate reactions, namely, poly(A) tailing, adapter ligation, reverse transcription, and finally a miR-Amp reaction. The poly(A) tailing reaction involved the addition of a 3`-adenosine tail to the miRNA, catalysed by the enzyme poly(A) polymerase. 2μl of each RNA sample was aliquoted into individual wells of a MicroAmp^™^ Optical 96-Well Reaction Plate. A reaction mix was then prepared with the poly(A) tailing reagents, as per the manufacturer’s protocol, after which, 3μl transferred into each well of the plate. The reaction plate was sealed with adhesion film, mixed and centrifuged. Following that, the plate was incubated in a QuantStudio^™^ 7 Flex Real-Time PCR System (Applied Biosystems, Life Technologies Corporation, Johannesburg, South Africa). The following settings were used to configure the QuantStudio^™^ 7 Flex – polyadenylation at 37°C for 45min, then a stop reaction at 65°C for 10min and finally an infinite hold step at 4°C. Following incubation, the plate was removed and the adapter ligation step commenced. The miRNA with poly(A) tails underwent adaptor ligation at their 5` end. In accordance with manufacturer guidelines, a new reaction mix was prepared with the adapter ligation reagents, following that 10μl of the mix was transferred into each well of the reaction plate containing the poly(A) tailing reaction product. After sealing the plate, it was briefly mixed, centrifuged and then incubated in the QuantStudio^™^ 7 Flex using the following settings – ligation at 16°C for 60min, followed by an infinite hold step at 4°C. The miRNA was then reverse-transcribed into cDNA. This reaction entailed the binding of a universal RT primer to the 3`-poly(A) tails of the miRNA. Sufficient reaction mix was prepared and 15μl added to each well of the 96-well reaction plate containing the adapter ligation reaction products. After sealing the plate, it was briefly mixed, centrifuged and incubated – reverse transcription at 42°C for 15min, then a stop reaction at 85°C for 5min, followed by an infinite hold at 4°C. The miR-Amp reaction step then followed, in which universal forward and reverse primers amplified the number of cDNA templates present in each sample. The reaction mix was prepared as per manufacturer specifications and 45μl transferred into a new 96-well reaction plate. A total volume of 5μl of the reverse transcription reaction product from the previous reaction step was added to each well of the new plate containing the miR-Amp reaction mix and incubation commenced at the following cycling conditions – enzyme activation at 95°C for 5min, for 1cycle, then denaturation at 95°C for 3s, for 14cycles, thereafter annealing/extension at 60°C for 30s, for 14cycles, a stop reaction at 99°C for 10min, for 1cycle, and a hold step at 4°C, for 1cycle. After successful completion of cDNA synthesis, samples were stored at −20°C until required for reverse transcriptase qPCR (RT-qPCR) analysis.

Prior to performing RT-qPCR, resultant cDNA samples were diluted 1:10 for optimum quantitative analysis. Thereafter, miRNA expression levels were evaluated, as per manufacturer instructions, using pre-designed TaqMan Advanced miRNA Assay primers for the investigated miRNAs: miR-30a-5p (assay ID: 479448_mir; catalogue number: A25576), miR-1299 (assay ID: 478696_mir; catalogue number: A25576), miR-182-5p (assay ID: 477935_mir; catalogue number: A25576), miR-30e-3p (assay ID: 478388_mir; catalogue number: A25576) and miR-126-3p (assay ID: 477887_mir; catalogue number: A25576; Applied Biosystems, Thermo Fisher Scientific, Johannesburg, South Africa). Data were obtained as Ct values and normalized to an endogenous control, miR-16-5p (assay ID: 477860_mir; catalogue number: A25576; Applied Biosystems, Johannesburg, Thermo Fisher Scientific, South Africa). Minimal differences were observed in the expression of the endogenous control between varied glycaemic groups, validating its stability, particularly between newly diagnosed vs. treated diabetes (Weale et al., 2020). The 2^−ΔCt^ method was used to evaluate the miRNA expression level in each sample, whilst the 2^−ΔΔCt^ value was used as the measure of the miRNA expression in each sample analysed compared with the control sample (Livak and Schmittgen, 2001).

### Statistical Analysis

Analysis of data was ++performed using SPSS v.25 (IBM Corp, 2011). The data were tested for normality using Normal Q-Q Plots. The results for categorical variables were presented as count (and percentages), whilst continuous variables were presented as mean (and standard deviation) for normally distributed variables, and median with 25th–75th percentiles was presented for skewed distributions. For comparison between glucose tolerance groups, analysis of the (ANOVA) and the Kruskal-Wallis test were used for continuous variables, whilst the chi-square test was used to assess categorical variables. Spearman’s partial correlations, adjusted for age, sex and body mass index (BMI), were performed to assess the relationship between miRNA expression and other variables. Furthermore, multivariate regression analysis was conducted in order to assess differences in miRNA expression with duration of disease. Various models were used, which were Model 1: Crude; Model 2: adjusted for age and sex; Model 3: adjusted for age, sex and type of medication; and Model 4: adjusted for adjusted for age, sex and type of medication, serum total cholesterol (cholesterol-S), usCRP, log gamma GT-S and log S-creatinine. A *value of p* < 0.05 was used to characterize statistically significant results.

## Results

### Basic Characteristics of Participants

As illustrated in Table 1, participants were 53.8years old on average, with the majority being female (72.7%), and most being normotolerant (*n*=974). As expected, glycaemic parameters (fasting blood glucose, 2-h blood glucose and HbA1c) were significantly higher in the screen-detected and known diabetes groups vs. the normotolerant group (*p*<0.001). Body mass index (BMI), waist circumference and hip circumference were significantly higher in both screen-detected DM and known DM, compared to the normotolerant group (*p*<0.001). Additionally, lipid variables, such as triglycerides-S and cholesterol LDL-S, increased significantly across the glycaemic groups (*p*<0.001), whilst cholesterol HDL-S exhibited a significant reduction from the normotolerant group through to the known DM group (*p*=0.039). Both systolic blood pressure and diastolic blood pressure measurements were observed to increase significantly across the glycaemic groups (all of which, *p*<0.001). Inflammatory markers usCRP and gamma GT-S were significantly higher in the diabetic groups in contrast to the normotolerant group, with reduced levels in known DM vs. screen-detected. Of the known diabetics on treatment, 165 (87.8%) were on oral medication, whilst 23 (12.2%) were on either both oral and/or insulin treatment.

**Table 1:** Characteristics of participants according to diabetic status.

	Normal	Screened DM	Known DM	*Value of p*
	*n*=974	*n*=92	*n*=188	
miR-30a-5p(2-ΔCt)	0.0252 ± 0.0549	0.0494 ± 0.0916	0.1577 ± 0.2512	<0.001
miR-30a-5p (2-ΔCt)^*^	0.0086 (0.0023;0.0262)	0.0169 (0.0041;0.0576)	0.0664 (0.011;0.1858)	<0.001
miR-1299 (2-ΔCt)	0.0033 ± 0.0108	0.0042 ± 0.009	0.0264 ± 0.062	<0.001
miR-1299 (2-ΔCt)^*^	0.0007 (0.0001;0.0023)	0.0009 (0.0003;0.0036)	0.0046 (0.0012;0.0202)	<0.001
miR-182-5p (2-ΔCt)	1.457 ± 2.0279	2.4347 ± 4.742	8.979 ± 11.1365	<0.001
miR-182-5p (2-ΔCt)^*^	0.7994 (0.3065;1.7989)	1.3223 (0.3966;2.4185)	4.6079 (1.3219;13.0158)	<0.001
miR-30e-3p (2-ΔCt)	0.0047 ± 0.0071	0.0056 ± 0.0067	0.0291 ± 0.0478	<0.001
miR-30e-3p (2-ΔCt)^*^	0.0019 (0.0006;0.0062)	0.0023 (0.0008;0.0079)	0.0119 (0.0025;0.041)	<0.001
miR-126-3p (2-ΔCt)	1.0375 ± 0.9964	1.6141 ± 1.324	7.2226 ± 7.1824	<0.001
miR-126-3p (2-ΔCt)^*^	0.7251 (0.2917;1.5107)	1.4188 (0.4404;2.4671)	4.7558 (1.4091;10.7017)	<0.001
Age (years)	45.22 ± 15.3	58.15 ± 10.62	57.88 ± 11.97	<0.001
**Sex, n (%)**			
Female	688 (70.6)	73 (79.3)	151 (80.3)	0.008
Male	286 (29.4)	19 (20.7)	37 (19.7)	
Body mass index (kg/m^2^)	27.4 ± 7.8	31.5 ± 8.0	30.7 ± 6.4	<0.001
Waist circumference (cm)	87.9 ± 16.5	100.2 ± 15.5	99.3 ± 16.5	<0.001
Hip circumference (cm)	100.9 ± 16.5	108.0 ± 15.4	107.0 ± 14.0	<0.001
Systolic blood pressure (mmHg)	131 ± 25	146 ± 26	148 ± 26	<0.001
Diastolic blood pressure (mmHg)	84 ± 15	90 ± 14	88 ± 15	<0.001
Glucose (Fasting) (mmol/L)^*^	4.7 (4.4;5.1)	7.3 (5.7;8.9)	9.1 (6.6;13.3)	<0.001
Glucose 2h (mmol/L)^*^	5.4 (4.5;6.3)	12.8 (11.5;16.6)		<0.001^**^
HbA1c (%)	5.6 ± 0.5	7.3 ± 1.9	8.9 ± 2.4	<0.001
HbA1c (mmol/mol)	37.5 ± 5.0	56.5 ± 21.2	73.8 ± 26.6	<0.001
Insulin Fasting (mIU/L)^*^	5.8 (3.7;9.0)	9.4 (5.45;16.6)	9.25 (5.48;15.43)	<0.001
Insulin 2h (mIU/L)^*^	30.5 (15.9;53.6)	51.2 (29.2;80.4)		<0.001^**^
Triglycerides-S (mmol/L)^*^	1.1 (0.8;1.5)	1.4 (1.1;2.4)	1.6 (1.2;2.2)	<0.001
Cholesterol HDL-S (mmol/L)	1.4 ± 0.4	1.3 ± 0.5	1.3 ± 0.3	0.039
Cholesterol LDL-S (mmol/L)	3.1 ± 1,0	3.5 ± 1.1	3.2 ± 1.1	0.001
Cholesterol-S (mmol/L)	5.0 ± 1.2	5.7 ± 1.3	5.3 ± 1.2	<0.001
usCRP (mg/L)^*^	3.4 (1.3;7.8)	6.5 (3.2;13.1)	4.9 (2.3;10.3)	<0.001
Gamma GT-S ^*^ (IU/L)^*^	27.0 (19.0;42.0)	42.5 (26.3;76.0)	33.0 (20.0;61.0)	<0.001
S-Creatinine (umol/L)^*^	59.0 (52.0;68.0)	61.0 (52.0;77.0)	60.5 (52.0;75.0)	0.127
MDRD eGFR (ml/min)	107.3 ± 30.0	93.2 ± 30.7	93.9 ± 32.5	<0.001
CKD-EPI eGFR (ml/min)	106.3 ± 20.6	90.3 ± 23.1	90.8 ± 24.1	<0.001
**Education level, n (%)**			
<7years	271 (28)	39 (43.3)	75 (40.3)	<0.001
≥7years	698 (72)	51 (56.7)	111 (59.7)	
**Tobacco use, n (%)**			
Non-smoker	393 (42.1)	58 (66.7)	128 (70.3)	<0.001
Current smoker	540 (57.9)	29 (33.3)	54 (29.7)	
**Alcohol use, n (%)**			
Non-drinker	645 (66.5)	76 (83.5)	161 (86.6)	<0.001
Current drinker	325 (33.5)	15 (16.5)	25 (13.4)	

*Cholesterol HDL-S, serum high-density lipoprotein cholesterol; cholesterol LDL-S, serum low-density lipoprotein cholesterol; cholesterol-S, serum total cholesterol; usCRP, ultrasensitive C-reactive protein; gamma GT-S, serum gamma-glutamyl transferase; S-creatinine, serum creatinine; MDRD eGFR, Modification of Diet in Renal Disease estimated glomerular filtration rate; and CKD-EPI eGFR, Chronic Kidney Disease Epidemiology Collaboration estimated glomerular filtration rate*.

* data represented as median (25th–75th percentile).

** value of p between normal group and screen-detected diabetes only.

### Relative miRNA Expression

**Figure 1:**
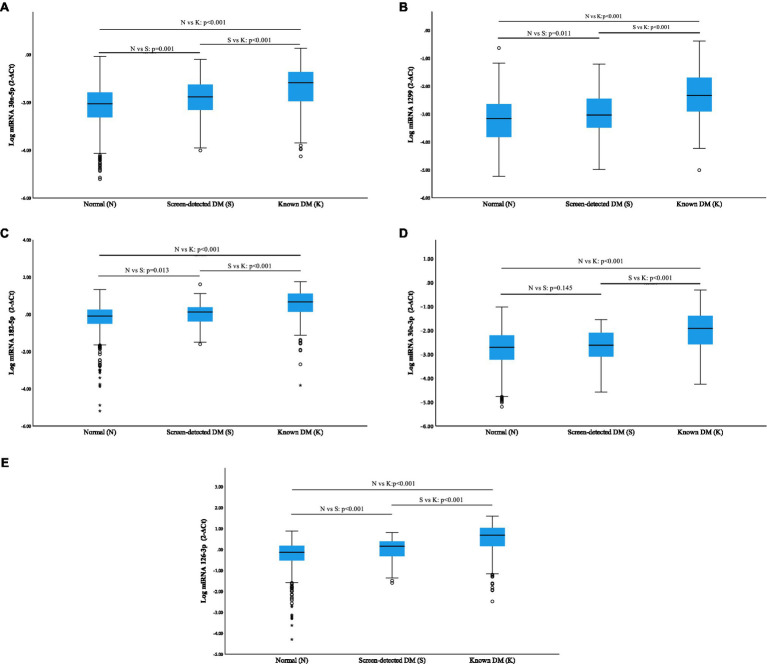
Relative expression of miRNAs according to glycaemic status using log miRNA 2^−ΔCt^. Normalization was relative to the expression of miR-16-5p. Data are shown as median (25th–75th percentile). **(A)** miR-30a-5p; **(B)** miR-1299; **(C)** miR-182-5p; **(D)** miR-30e-3p; and **(E)** miR-126-3p.

All miRNAs were significantly overexpressed in subjects with known diabetes when compared to normotolerant individuals (*p*<0.001), and additionally, a significant elevation was observed in known diabetics vs. screen-detected (*p*<0.001; Figure 1). miR-30a-5p, miR-1299, miR-182-5p, miR-30e-3p and miR-126-3p were all significantly upregulated in screen-detected DM compared to the normotolerant group (*p*≤0.013), with the exception of miR-30e-3p (*p*=0.145), and miR-30a-5p exhibited the most significant increase in expression (*p*=0.001) between the two groups.

### Comparisons Between miRNA Expression With Treatment and Duration of Disease

The median duration of condition in individuals with known diabetes and on anti-diabetic treatment was 8years. Although not significant, all other miRNAs with the exception of miR-182-5p were increased in individuals who had had diabetes for over 8years with miR-30e-3p and miR-126-3p nearing significance, *p*≤0.095. miR-182-5p was significantly reduced in individuals who had had diabetes for over 8years, *p*=0.033 (Table 2). No significant differences were observed between the expression of miRNAs and the type of treatment, that is, oral or combination of oral and/or insulin, *p*>0.05 (Table 2).

**Table 2:** Comparisons between miRNA expression with treatment type and duration of disease.

	Types of medication			Duration of disease		
	Both/Insulin	Oral	*value of p*	<8years	≥8years	*value of p*
miR-30a-5p (2^–wΔCt^)	0.0641 (0.0292; 0.2787)	0.0664 (0.0098; 0.184)	0.280	0.0581 (0.0069; 0.1787)	0.0607 (0.0182; 0.1906)	0.567
miR-1299 (2^–ΔCt^)	0.0048 (0.0015; 0.0233)	0.0045 (0.0011; 0.0196)	0.665	0.0041 (0.0006; 0.0207)	0.0075 (0.0015; 0.0243)	0.155
miR-182-5p (2^–ΔCt^)	7.4965 (2.183; 14.1241)	4.5773 (1.1516; 12.9642)	0.427	3.2669 (1.0255; 10.3437)	6.4257 (2.2623; 13.4441)	0.033
miR-30e-3p (2^–ΔCt^)	0.0098 (0.003; 0.0375)	0.0128 (0.0025; 0.0431)	0.765	0.0073 (0.0019; 0.0295)	0.0128 (0.0034; 0.0437)	0,065
miR-126-3p (2^–ΔCt^)	4.1712 (1.765; 9.1078)	4.8205 (1.3649; 10.9543)	0.873	3.8398 (1.3521; 8.4545)	5.749 (2.0933; 11.674)	0.095

### Partial Spearman’s Correlation Coefficients Adjusted for Age, Sex and Body Mass Index

Spearman’s non-parametric correlations, adjusted for age, sex and BMI, were conducted in order to identify significant associations between the expression of the selected miRNAs and other parameters (Table 3). All miRNAs observed significant positive correlations with each other (all, *p*≤0.002), with the exception of miR-1299 and miR-126-3p (*p*=0.075). Correlations with glucose indices revealed significant negative correlations between miR-30a-5p and miR-30e-3p with fasting blood glucose (*r*=−0.308, *p*=0.045 and *r*=−0.315, *p*=0.040, respectively). Likewise, miR-30e-3p demonstrated a significant negative correlation with HbA1c, *r*=−0.318, *p*=0.037. The other target miRNAs, however, did not observe noteworthy correlations with any of the abovementioned glycaemic indices. With respect to obesity markers, only miR-30a-5p and miR-30e-3p correlated significantly and negatively, with triglycerides-S (*r*=−0.319, *p*=0.037 and *r*=−0.371, *p*=0.014, respectively), whilst the remaining miRNAs demonstrated no such associations. All miRNAs exhibited significant negative correlations with cholesterol HDL-S (all of which, *p*≤0.046), whilst none demonstrated any significant links with cholesterol LDL-S. Although, with respect to cholesterol-S, all miRNAs, with the exception of miR-1299, showed notable negative correlations, all of which were *p*≤0.036. Correlations with known inflammatory markers revealed significant negative associations between all miRNAs and both usCRP and gamma GT-S (all, *p*≤0.046); miR-1299, however, did not observe any significant correlation with gamma-GT-S. Additionally, all miRNAs demonstrated significant correlations with markers of kidney function, namely, S-creatinine, MDRD eGFR (Modification of Diet in Renal Disease estimated glomerular filtration rate) and CKD-EPI eGFR (Chronic Kidney Disease Epidemiology Collaboration estimated glomerular filtration rate), all of which were *p*≤0.047.

**Table 3:** Partial Spearman’s correlation coefficients adjusted for age, sex and body mass index (BMI).

	miR-30a-5p (2^–ΔCt^)		miR-1299 (2^–ΔCt^)		miR-182-5p (2^–ΔCt^)		miR-30e-3p (2^–ΔCt^)		miR-126-3p (2^–ΔCt^)	
*Adjusted for age, BMI and sex*	r	*value of p*	r	*value of p*	r	*value of p*	r	*value of p*	r	*value of p*
miR-30a-5p (2^–ΔCt^)	1.000	–	0.497	0.001	0.739	<0.001	0.668	<0.001	0.587	<0.001
miR-1299 (2^–ΔCt^)	0.497	0.001	1.000	–	0.582	<0.001	0.452	0.002	0.275	0.075
miR-182-5p (2^–ΔCt^)	0.739	<0.001	0.582	<0.001	1.000	–	0.488	0.001	0.620	<0.001
miR-30e-3p (2^–ΔCt^)	0.668	<0.001	0.452	0.002	0.488	0.001	1.000	–	0.562	<0.001
miR-126-3p (2^–ΔCt^)	0.587	<0.001	0.275	0.075	0.620	<0.001	0.562	<0.001	1.000	–
Waist circumference	−0.078	0.618	−0.002	0.988	−0.134	0.392	−0.067	0.670	−0.200	0.198
Hip circumference (cm)	−0.076	0.628	0.000	0.998	−0.130	0.406	−0.065	0.681	−0.197	0.205
Systolic blood pressure (mmHg)	−0.173	0.266	−0.164	0.293	−0.227	0.143	−0.151	0.335	−0.164	0.292
Diastolic blood pressure (mmHg)	−0.155	0.320	−0.149	0.340	−0.206	0.186	−0.135	0.389	−0.150	0.338
Glucose (Fasting) (mmol/L)	−0.308	0.045	−0.207	0.183	−0.227	0.144	−0.315	0.040	−0.247	0.110
HbA1c (%)	−0.260	0.093	−0.247	0.110	−0.247	0.111	−0.318	0.037	−0.232	0.134
Insulin Fasting (mIU/L)	−0.171	0.273	−0.181	0.245	−0.200	0.198	−0.221	0.154	−0.211	0.175
Triglycerides-S (mmol/L)	−0.319	0.037	−0.281	0.068	−0.297	0.053	−0.371	0.014	−0.280	0.069
Cholesterol HDL-S (mmol/L)	−0.381	0.012	−0.306	0.046	−0.326	0.033	−0.388	0.010	−0.312	0.041
Cholesterol LDL-S (mmol/L)	−0.297	0.053	−0.280	0.069	−0.281	0.068	−0.300	0.051	−0.222	0.153
Cholesterol-S (mmol/L)	−0.385	0.011	−0.293	0.056	−0.320	0.036	−0.434	0.004	−0.326	0.033
usCRP (mg/L)	−0.388	0.010	−0.306	0.046	−0.323	0.035	−0.435	0.004	−0.325	0.033
Gamma GT-S (IU/L)	−0.385	0.011	−0.294	0.056	−0.320	0.037	−0.434	0.004	−0.325	0.033
S-Creatinine (umol/L)	−0.390	0.007	−0.303	0.039	−0.328	0.025	−0.427	0.003	−0.328	0.024
MDRD eGFR (ml/min)	−0.383	0.011	−0.306	0.046	−0.318	0.038	−0.425	0.004	−0.319	0.037
CKD-EPI eGFR (ml/min)	−0.382	0.011	−0.305	0.047	−0.316	0.039	−0.424	0.005	−0.318	0.038

*Cholesterol HDL-S, serum high-density lipoprotein cholesterol; cholesterol LDL-S, serum low-density lipoprotein cholesterol; cholesterol-S, serum total cholesterol; usCRP, ultrasensitive C-reactive protein; gamma GT-S – serum gamma-glutamyl transferase; S-creatinine – serum creatinine; MDRD eGFR, Modification of Diet in Renal Disease estimated glomerular filtration rate; and CKD-EPI eGFR, Chronic Kidney Disease Epidemiology Collaboration estimated glomerular filtration rate*.

### Multivariate Regression Analysis of miRNAs for the Duration of Diabetes

The log miRNA 2^-ΔCt^ was used to perform multivariate regression analysis, in order to demonstrate the relationships between the expression of the investigated miRNAs and the duration of T2DM (Table 4). The expressions of miRNA-1,299, miR-182-5p and miR-126-3p were associated with duration of diabetes when the model was adjusted for age and sex [odds ratio (OR)≥0.076, 95% confidence interval (CI): 0.001–0.151, *p*≤0.046]; however, after adjustment for type of treatment, only the expression of miR-182-5p remained independently associated with the duration of the disease (OR: 0.127, CI: 0.018–0.236, *p*=0.023). Upon further adjustment for usCRP, log gamma GT-S and log S-creatinine, miR-182-5p expression retained significance in its association with duration of T2DM (OR: 0.145, CI: 0.034–0.255, *p*=0.011); moreover, miR-30e-3p as well as miR-126-3p also exhibited notable associations [(OR: 0.103, CI: 0.007–0.199, *p*=0.036) and (OR: 0.145, CI: 0.020–0.270, *p*=0.024), respectively].

**Table 4:** Univariate regression analysis of miRNAs for the duration of diabetes.

	B	Std error	95% Confidence Interval (CI)		*value of p*
			Lower	Upper	
**Log miR-30a-5p (2^–ΔCt^)**					
Model 1	−0.021	0.037	−0.094	0.053	0.581
Model 2	0.023	0.036	−0.047	0.094	0.511
Model 3	0.029	0.040	−0.049	0.108	0.464
Model 4	0.049	0.041	−0.032	0.130	0.234
**Log miR-1299 (2^–ΔCt^)**					
Model 1	0.058	0.040	−0.022	0.138	0.151
Model 2	0.076	0.038	0.001	0.151	0.046
Model 3	0.064	0.027	−0.010	0.138	0.088
Model 4	0.068	0.038	−0.007	0.142	0.075
**Log miR-182-5p (2^–ΔCt^)**					
Model 1	0.078	0.040	0.000	0.156	0.051
Model 2	0.101	0.037	0.029	0.174	0.007
Model 3	0.127	0.055	0.018	0.236	0.023
Model 4	0.145	0.056	0.034	0.255	0.011
**Log miR-30e-3p (2^–ΔCt^)**					
Model 1	0.040	0.045	−0.050	0.130	0.384
Model 2	0.070	0.042	−0.013	0.153	0.097
Model 3	0.086	0.047	−0.008	0.179	0.072
Model 4	0.103	0.048	0.007	0.199	0.036
**Log miR-126-3p (2^–ΔCt^)**					
Model 1	0.083	0.043	−0.012	0.177	0.086
Model 2	0.102	0.044	0.014	0.189	0.023
Model 3	0.119	0.062	−0.003	0.241	0.056
Model 4	0.145	0.063	0.020	0.270	0.024

*Model 1 Crude; Model 2: adjusted for Age and Sex; Model 3: adjusted for Age, Sex, Type of Medication; and Model 4: adjusted for Age, Sex, Type of Medication, Cholesterol-S, usCRP, log Gamma GT-S and log S-creatinine*.

## Discussion

In this study, the expressions of circulating miRNAs (miR-30a-5p, miR-1299, miR-182-5p, miR-30e-3p and miR-126-3p) were investigated in newly diagnosed and known diabetic individuals on treatment. Our data show that these miRNAs are differentially expressed in individuals with diabetes, as well as between newly diagnosed and those on treatment. All target miRNAs correlated significantly with each other, with the exception of miR-1299 and miR-126-3p. Correlations were illustrated between miR-30a-5p and miR-30e-3p with fasting blood glucose and HbA1c, as well as with triglycerides-S. Furthermore, all miRNAs, except miR-1299, correlated significantly with HDL-cholesterol and serum total cholesterol, as well as with inflammatory markers (usCRP and γ-glutamyltransferase), with similar associations exhibited with kidney function indicators, MDRD eGFR and CKD-EPI eGFR. We observed that the duration of the disease is the most determining factor in the expression of these miRNAs. For instance, miR-182-5p was significantly decreased in individuals who had diabetes for over 8years, but no such differences were observed when anti-diabetic treatment was taken into account. Moreover, miR-1299, miR-182-5p and miR-126-3p were significantly associated with T2DM in regression analysis adjusted for age and sex, but when medication was included in the model, that association was lost for miR-1299 and miR-126-3p and only retained by miR-182-5p.

Advances in whole-genome sequencing as well as epigenome profiling technologies have contributed toward accelerated growth in research aimed at unveiling the roles of epigenetics in human disease (Hawkins et al., 2010; Laird, 2010; Afjeh and Ghaderian, 2013; Koperski et al., 2017) particularly the roles of miRNAs in DM (Zampetaki et al., 2010; Karolina et al., 2011; Jiménez-Lucena et al., 2018a). miRNAs target-specific messenger RNA (mRNA), hindering translation of the subsequent proteins the mRNA encode for (Bartel, 2009), as such, understanding these potential targets and the resultant knock-on effects of altered miRNA expression, is essential in unravelling the intricate subclinical molecular mechanisms that drive disease development. For instance, a previous rat-based study demonstrated miR-30a-5p to be a mediator of beta-cell dysfunction-induced glucotoxicity, by suppressing the expression of Beta2/NeuroD, a gene responsible for regulating pancreatic beta-cell maintenance and insulin gene transcription (Noguchi et al., 2005; Kim et al., 2013). The investigation further established that an overexpression of the miRNA led to pancreatic beta-cell dysfunction (Kim et al., 2013). Additionally, in a follow-up study spanning 5years, Jiménez-Lucena and co-workers assessed plasma miRNAs, *via* RT-qPCR, in non-diabetic patients, in order to ascertain whether the combined use of these miRNAs with clinical and biochemical measures was more beneficial in predicting type 2 diabetes (Jiménez-Lucena et al., 2018b). Nine dysregulated miRNAs were identified, which when added to HbA1c, yielded acceptable predictive values in early detection of type 2 diabetes, one of which was miR-30a-5p. Elevated expression of the miRNA was found to be associated with increased risk of T2DM development, with higher levels of miR-30a-5p observed in participants diagnosed with T2DM in the first year of follow-up, in comparison with those diagnosed in later years. These findings were consistent with ours, in that we also observed marked miR-30a-5p expression in newly diagnosed diabetics vs. the normotolerant; however, the cross-sectional nature of our investigation thwarted us in accurately assessing the effects of prolonged disease on its expression.

Another promising candidate, miR-126-3p, has been heralded as a potential biomarker for early detection of dysglycaemia (Zampetaki et al., 2010; Zhang et al., 2013; Rezk et al., 2016) and its role as a regulator of angiogenesis is known (Wang et al., 2008). A recent report has shown insulin receptor substrate 1 (IRS1) as a target for miR-126-3p, demonstrating overexpression of the miRNA mediating marked reduction in IRS1 in smooth muscle cells. IRS1 plays an essential role in insulin signalling in hyperglycaemic states; thus, a reduction in its expression negatively impacts glucose metabolism (Tryggestad, 2019). miR-126-3p expression has also been investigated in complications associated with long-term diabetes, such as diabetic kidney disease (DKD; KDIGO, 2013; Bijkerk et al., 2015; Assmann et al., 2018). Bijkerk et al. (2015) determined significantly increased expression of circulating miR-126-3p in the plasma of subjects with diabetic nephropathy (with a mean duration of diabetes of 29years), in comparison with those without (Bijkerk et al., 2015). In accordance with these findings, a separate systematic review, which aimed at identifying studies comparing miRNA expression in persons with DKD and diabetics without the complication, showed that of the studies included, miR-126-3p was found to be overexpressed in either whole blood, plasma, urine or kidney tissue in patients with DKD (Assmann et al., 2018). These reports may offer a plausible explanation for our findings of altered miR-126-3p expression in diabetic states (both screen-detected and long-term diabetes), as well as the significant associations demonstrated with known measures of renal dysfunction. Although, in our sample, the median duration of diabetes was less, the findings of Bijkerk et al. (2015) corroborate the moderate associations we observed between dysregulated miR-126-3p and duration of diabetes. Moreover, Prattichizzo et al aimed to identify specific miRNA signatures from plasma extracellular vesicles (EVs) of diabetic individuals with and without complications (Prattichizzo et al., 2021b). These EVs are minute membranous particles that have been shown to transport miRNAs, with postulations that they may elucidate inter-tissue communications in disease states, through the expression patterns of the miRNA signatures they shuttle (Guay and Regazzi, 2017; Xiao et al., 2019). Prattichizzo and colleagues identified miRNAs influenced by diabetes, one of which was miR-126-3p; however, the miRNA did not witness significant differential expression in diabetics with complications (mean duration of disease being 20years). In our study, miR-126-3p expression observed similar susceptibility to dysglycaemic states; furthermore, although we do not report data pertaining to diabetic complications, we did however demonstrate moderate relationships between long-term T2DM (median duration of 8years) and its expression. In a similarly study, which included a treatment aspect, significantly decreased miR-126-3p levels were found in untreated diabetics as well as diabetics on metformin treatment, in comparison with levels in the control group (Ghai et al., 2019). We, however, did not witness any notable associations when treatment was accounted for. In light of these findings, it is relevant to mention that whole blood, which was the starting sample in our study, contains collective miRNAs from circulating plasma-specific EVs, blood-cell-derived miRNAs, as well as those bound to carriers such as HDL and RNA-binding proteins (Prattichizzo et al., 2021b). Pre-analytical centrifugation of samples is necessary for retrieving plasma from whole blood, and this may influence the expression profiles of miRNAs, due to the removal of blood-cell-specific miRNAs (Felekkis and Papaneophytou, 2020). All things considered, although whole blood provides better yields of miRNA, caution should still be approached when cross-comparing studies, due to the expression variations between tissues (Prattichizzo et al., 2021b).

Reports have also described the involvement of candidate miRNA, miR-182, in regulating glucose homeostasis, chiefly by targeting FOXO1 (Karolina et al., 2011; Zhou et al., 2014; Zhang et al., 2016). Mammalian cells express four FOXO variants, namely, FOXO1, FOXO3, FOXO4 and FOXO6, of which FOXO1 is the most abundantly expressed in liver, adipose tissue as well as in pancreatic beta-cells (Kitamura et al., 2002). FOXO1 is vital in regulating pancreatic beta-cell replication and differentiation, as well as maintenance in states of metabolic stress (Kitamura, 2013). Furthermore, FOXO1 is involved in stimulating hepatic gluconeogenesis in states of hypoglycaemia *via* the phosphoinositide-3-kinase/protein kinase B (PI3K/Akt) signalling pathway, whilst in hyperglycaemia, insulin signalling *via* insulin, like growth factor-1 (IGF-1) and its receptor (IGF-1R), stimulates PI3K/Akt-dependent phosphorylation of FOXO1, leading to subsequent suppression of gluconeogenesis (Tsuchiya and Ogawa, 2017). In this regard, in early stages of dysglycaemia, increased levels of miR-182 in individuals with prediabetes compared to those with newly diagnosed diabetes have been linked with attempted inhibition of hepatic gluconeogenesis, whilst pathological downregulation in diabetes promoted gluconeogenesis (Karolina et al., 2011). With respect to the direct impact of long-term hyperglycaemia and treatment, there is a paucity of information relating to miR-182-5p expression. Albeit, in a cancer-related study, Li et al. (2012) aimed at evaluating miRNAs connected with the anti-tumour effects of metformin in human gastric cancer cells. Upregulation of miR-182 was revealed in the cultured cells and cancer tissues treated with metformin when compared to untreated cells (Li et al., 2012). In our study, we did not observe any influences of anti-diabetic treatment (oral metformin and/or insulin) on the expression of the selected miRNAs, but rather marked differences with duration of the disease, especially with respect to miR-182-5p. miR-182-5p has, however, demonstrated links with diabetes-related complications, such as DKD, which is a common complication of diabetes (Ming et al., 2019). Ming and co-workers illustrated overexpression of miR-182-5p in the podocytes of individuals with DKD, as opposed to non-diabetic controls, alluding that this increased expression was linked to a reduction in CD2-associated protein, which is crucial in podocyte apoptosis and subsequent development of chronic kidney disease in diabetic persons (Ming et al., 2019). Ming et al. (2019) did not include information pertaining to the duration of diabetes in their study subjects, however, their findings corroborating the associations we found between miR-182-5p expression and key indicators of kidney function. Based on literature, our study mirrors previous findings in that miR-182-5p expression is indeed altered in hyperglycaemic states, though the underlying molecular mechanisms remain unclear. Furthermore, in light of the scarcity of reports pertaining to long-term T2DM and miR-182-5p expression, we have shown that the time span of disease in known diabetics has more of an influence on the expression of the miRNA, as opposed to treatment. This justifies the need for further research ventures, in order to ascertain these associations, particularly in diabetics with complications.

This is the first study of its kind to be conducted in an African setting, and findings may contribute toward curbing the increasing burden of T2DM in Africa. The study was, however, limited by the disproportionate representation of normotolerant vs. screen-detected diabetics and known diabetics on treatment. Additionally, the cross-sectional nature of the study limits accurate evaluation of anti-diabetic induced epigenetic changes; hence, longitudinal studies are advised. The absence of information pertaining to diabetes-associated complications in known diabetics hampered our ability to comprehensively assess the influences of long-term diabetes on miRNA expression, as such, it would be more beneficial to include such data in future. A further limitation was that the expression of the target miRNAs was assessed, without predicting and measuring potential target mRNA levels. Hence, future recommendations would be to explore and measure potential target mRNA levels to elucidate the knock-on effects of altered miRNA expression on downstream proteins. This would aid in more accurate comparisons of the underlying molecular interplays between controlled and uncontrolled diabetics.

## Conclusion

Our study has revealed important associations and altered expression patterns of miR-30a-5p, miR-1299, miR-182-5p, miR-30e-3p and miR-126-3p in individuals with diabetes on anti-diabetic treatment compared to newly diagnosed cases. Furthermore, we show that miR-182-5p in particular decreases with increasing duration of T2DM. Longitudinal and functional investigations are recommended to elucidate the involvement of the miRNA in insulin signalling and glucose homeostasis, to endorse its use as a therapeutic target in DM and its associated complications.

## Data Availability Statement

The datasets presented in this article are not readily available because of the terms of consent to which participants agreed, but are available from the principal investigator of the main study on reasonable request. Requests to access the datasets should be directed to TM, matshat@cput.ac.za.

## Ethics Statement

This study involving human participants was reviewed and approved by the Research Ethics Committees of the Cape Peninsula University of Technology and Stellenbosch University (NHREC: REC—230,408–014 and N14/01/003, respectively). Ethical clearance for this sub-study was sought from and granted by the Cape Peninsula University of Technology Ethics Committee (CPUT/HW-REC 2019/H3). The patients/participants provided their written informed consent to participate in this study.

## Author Contributions

All authors contributed significantly to this project. CW: wrote the first draft, experimental procedures, data analysis and interpretation. DM: experimental procedures, data analysis and interpretation. SD: recruitment and screening of cohort, statistical analysis and interpretation of data. SR: interpretation of data, editing and revising it for intellectual content. RE: conception, interpretation of the data, revising it for intellectual content and final approval of the version to be published. AK: conception, interpretation of the data, revising it for intellectual content and final approval of the version to be published. GD: editing and revising it for intellectual content and final approval of the version to be published. TM: conception and design of the study, analysis and interpretation of the data, revising it for intellectual content and final approval of the version to be published.

## Funding

This research project was supported by grants from the South African Medical Research Council (SAMRC), with funds from National Treasury under its Economic Competitiveness and Support Package (MRC-RFA-UFSP-01-2013/VMH Study), South African National Research Foundation (SANRF) grant no. 115450). Any opinions, findings, conclusions or recommendations expressed in this article are those of the authors, and the SAMRC and/or SANRF do not accept any liability in this regard.

## Conflict of Interest

The authors declare that the research was conducted in the absence of any commercial or financial relationships that could be construed as a potential conflict of interest.

## Publisher’s Note

All claims expressed in this article are solely those of the authors and do not necessarily represent those of their affiliated organizations, or those of the publisher, the editors and the reviewers. Any product that may be evaluated in this article, or claim that may be made by its manufacturer, is not guaranteed or endorsed by the publisher.
